# The Reverse Warburg Effect Is Associated with Fbp2-Dependent Hif1α Regulation in Cancer Cells Stimulated by Fibroblasts

**DOI:** 10.3390/cells9010205

**Published:** 2020-01-14

**Authors:** Przemysław Duda, Jakub Janczara, James A. McCubrey, Agnieszka Gizak, Dariusz Rakus

**Affiliations:** 1 Department of Molecular Physiology and Neurobiology, University of Wrocław, Sienkiewicza 21 Street, 50-335 Wrocław, Poland; przemyslaw.duda@uwr.edu.pl (P.D.); jakub.janczara@upwr.edu.pl (J.J.); agnieszka.gizak@uwr.edu.pl (A.G.); 2 Department of Biochemistry and Molecular Biology, Wrocław University of Environmental and Life Sciences, Norwida 31 Street, 50-375 Wrocław, Poland; 3 Department of Microbiology and Immunology, Brody School of Medicine at East Carolina University, 600 Moye Boulevard, Greenville, NC 27858, USA; mccubreyj@ecu.edu

**Keywords:** fructose 1,6-bisphosphatase 2, glycolysis, hypoxia-inducible factor-1α, lung cancer

## Abstract

Fibroblasts are important contributors to cancer development. They create a tumor microenvironment and modulate our metabolism and treatment resistance. In the present paper, we demonstrate that healthy fibroblasts induce metabolic coupling with non-small cell lung cancer cells by down-regulating the expression of glycolytic enzymes in cancer cells and increasing the fibroblasts’ ability to release lactate and thus support cancer cells with energy-rich glucose-derived metabolites, such as lactate and pyruvate—a process known as the reverse Warburg effect. We demonstrate that these changes result from a fibroblasts-stimulated increase in the expression of fructose bisphosphatase (Fbp) in cancer cells and the consequent modulation of Hif1α function. We show that, in contrast to current beliefs, in lung cancer cells, the predominant and strong interaction with the Hif1α form of Fbp is not the liver (Fbp1) but in the muscle (Fbp2) isoform. Since Fbp2 oligomerization state and thus, its role is regulated by AMP and NAD^+^—crucial indicators of cellular metabolic conditions—we hypothesize that the Hif1α-dependent regulation of the metabolism in cancer is modulated through Fbp2, a sensor of the energy and redox state of a cell.

## 1. Introduction

It has long been recognized that tumor metabolism relies on the glycolytic production of ATP despite the presence of oxygen [1], and this type of metabolism is known as the Warburg effect. 

The molecular basis of the Warburg effect has been clarified by Vander Heiden et al. [2] who demonstrated that cancer cells use pyruvate kinase 2 (Pkm2) to short-circuit ATP production and avoid the inhibition of glycolysis. The elevated expression of glycolytic enzymes in cancers has been shown to rely on the increased stability of hypoxia inducible factor 1 α (Hif1α), which is the master transcriptional regulator of glycolysis. Hif1α is stabilized by Pkm2 while fructose 1,6-bisphosphatase 1 (Fbp1, the liver isozyme of Fbp) leads Hif1α to degrade (respectively, [3,4]).

However, during the last decade, several lines of evidence have accumulated to suggest that, in some cancers, the elevated glycolysis and the Warburg effect may be attributed to tumor stromal but not cancer cells [5]. In this scenario, called the reverse Warburg effect, stromal cells (e.g., cancer-associated fibroblasts; CAFs) support cancer cells metabolism by releasing glucose-derived metabolites, such as lactate and pyruvate (for review see: [6,7]).

While the effects of CAFs on cancer cells metabolism have been intensively studied [5,8,9,10,11], much less data is available on the metabolic cross-talk between healthy fibroblasts and cancer cells. 

Fibroblasts belong to the most abundant cells of the tumor microenvironment, and they have a great impact on tumorigenesis by contributing to the cancer cells proliferative capacity, the response to cell death signaling, the induction of angiogenesis and cancer-associated inflammation, and reprogramming energy metabolism (for review see: [12,13]).

Recently, it has been shown that the co-culturing of human lung fibroblasts with the H1299 human lung cancer cell line stimulated the expression of mitochondrial biogenesis proteins in the H1299 cells. At the same time, the co-culturing elevated expression of mRNAs for several glycolysis-related genes and reduced oxidative phosphorylation in fibroblasts [10]. These changes, consistent with the reverse Warburg effect, raised the following question: is the shift from glycolytic to oxidative metabolism in cancer cells associated with an elevation of Fbp and a decrease of Hif1α levels?

The results presented here demonstrate that healthy mouse and human fibroblasts regulate the expression of proteins involved in the Warburg effect, such as hexokinase 2 (Hk2) and lactate dehydrogenase A (Ldha) in murine and human non-small cell lung carcinoma cell lines—the KLN205 and A549 line, respectively. These fibroblasts-induced changes are associated with a decrease in the Hif1α level, which correlates with an elevation in the Fbp expression in the cancer cells.

Moreover, our study demonstrates that in lung cancer cells, the predominant form of Fbp is Fbp2, not Fbp1, and that Hif1α interacts with Fbp2 much more strongly than with Fbp1.

The Fbp2 oligomerization state, and thus its role/interactions in a cell, is regulated by AMP and NAD^+^ (for review see: [14]), both of which are crucial indicators of cellular metabolic conditions. Therefore, we hypothesize that the hypoxia/Hif1α-dependent regulation of the metabolism in cancer is modulated through Fbp2, and is based on the energy and redox state of a cell.

## 2. Materials and Methods

### 2.1. Cell Isolation and Culture

Murine lung fibroblasts (MLF) were isolated from newborn BALB/c mice according to Zilberberg et. al. [15]. The protocol of isolation was approved by the II Local Scientific Research Ethical Committee, Wroclaw University of Environmental and Life Sciences. Briefly, lungs were cut into small pieces and digested for 90 min at 37 °C with 2.4 U/mL Dispase II (Roche, Basel, Switzerland) and 0.1% Collagenase A (Roche). The digested tissue was washed twice in PBS with addition of 0.05 M EDTA, centrifuged at 1500× *g* for 5 min and plated in tissue culture dishes in a Minimum Essential Medium (MEM) supplemented with 10% FBS, 2 mM L-glutamine and 100 units/mL penicillin/streptomycin. The medium was changed after 1 h of culture growth. The cells were grown to 80% confluency before the freezing or passaging procedures.

The KLN205 cells (American Type Culture Collection, Old Town Manassas, VA, USA) and their co-cultures with the human lung fibroblasts (HLF) (Sigma-Aldrich, St. Louis, MO, USA, 506-05a) and MLF were seeded in a density of 6000 cells/cm^2^ and grown in MEM supplemented with 10% FBS, 2 mM L-glutamine, 1% non-essential amino acid solution (Sigma, M7145) and 100 units/mL penicillin/streptomycin. A549 cells (obtained from the Department of Human Morphology and Embryology at Wroclaw Medical University) and their co-cultures with the HLF were seeded in the same density of 6000 cells/cm^2^ and grown in the Dulbecco’s Modified Eagle’s Medium high glucose (Sigma, D6046) supplemented with 10% FBS and 100 units/mL penicillin/streptomycin. For the co-culture, cancer cells and fibroblasts were seeded onto one plate in the 1:1 ratio. All cells were cultured at 37 °C in a humidified atmosphere with 5% CO_2_ for 24–72 h.

### 2.2. Microvesicles Purification and Labeling

After 48 h with the HLF monoculture, the culture medium, from a 25 cm^2^ culture flask, was collected and microvesicles were purified according to the method described in [16]. To confirm their isolation and uptake by cells, the microvesicles were stained with a PKH67 Green Fluorescent Cell Linker Kit and were incubated for 24 h (as described in [17]) with the A549 monoculture. Then, the cells were fixed in 4% paraformaldehyde, permeabilized, and the immunofluorescent detection of Fbp was performed (as described in Section 2.4) and observed using a confocal microscope. Alternatively, the monoculture was treated for 24 h with unstained microvesicles or with the whole medium obtained from the HLF monoculture (“conditioned medium”), meaning that the supernatant would remain after the isolation procedure. Subcellular localization of Fbp was detected as described below (Section 2.4).

### 2.3. Fbp2 Expression Silencing

Fbp2 expression silencing in the KLN205 cells was carried out with MISSION^®^ shRNA Plasmid DNA (Merck, Kenilworth, NJ, USA, SHCLND-NM_007994). The transfection of plasmid DNA was performed using a Lipofectamine^TM^ 2000 Transfection Reagent (Invitrogen, Carlsbad, CA, USA, 11668) according to the protocol provided by the manufacturer. The amount of plasmid DNA used was 1.25 μg, and 2.5 μL of Lipofectamine^TM^ 2000 Transfection Reagent were used per well on a 12-well plate.

### 2.4. Immunofluorescence

The immunofluorescence studies were performed as described previously [18]. The cells were fixed, permeabilized, and incubated overnight at 4 °C with the following respective primary antibodies: rabbit anti-Fbp2 (1:500, produced and tested as described previously [19]), mouse anti-Fbp2 (1:500, Santa Cruz Biotechnology, Dallas, TX, USA, sc-271799), rabbit anti-Hif1α (1:500, Bioss Antibodies, Woburn, MA, USA, bs-0737R), rabbit anti-Ldha (1:500, Novus Biologials, Littleton, CO, USA, NBP1-48336), rabbit anti-Hk2 (1:500, Merck ab3279), rabbit anti-Ki67 (1:500, Abcam, Cambridge, UK, ab15580), mouse anti-αSMA (1:500, Merck a5228), mouse anti-β-actin (1:500, Sigma-Aldrich a1978), and rabbit anti-β-actin (1:500, Sigma–Aldrich a2066). The primary antibodies were detected using the following fluorophore-labelled secondary antibodies: goat anti-mouse-AlexaFluor488 (1:1000, Abcam, ab150113), goat anti-mouse-AlexaFluor633 (1:1000, Thermo Fisher Scientific, Waltham, MA, USA, a21050), goat anti-rabbit-AlexaFluor 488 (1:1000, Invitrogen a11034), and goat anti-rabbit-AlexaFluor633 (1:1000, Invitrogen a21070). Nuclei were counterstained with DAPI. In the controls, the primary antibodies were omitted.

### 2.5. In Situ Detection of Protein Interaction

The endogenous detection of Fbp2-Hif1α interaction was performed using the Duolink^®^ in situ Orange Starter Kit Mouse/Rabbit (Sigma-Aldrich). The procedure was performed according to the protocol provided by the manufacturer, using rabbit anti-Hif1α (1:500, Bioss, bs-0737R) and mouse anti-Fbp2 (1:500, Santa Cruz, sc-271799) primary antibodies. In the controls, the primary antibodies were omitted.

### 2.6. Fluorescent In Situ Hybridization (FISH)

FISH was performed as described previously [18]. The following oligonucleotides complementary to mouse mRNA sequences were used: Fbp1 (5′-[Cyanine5]GACGGGTCCA GCATGAAGCA GTTGACACCA CAATCC-3′), Fbp2 (5′-[Cyanine3]GCACACAGCT GAGATACTCT TGCACATCCT CAGGGGAC-3′), and Ldha (5′-[Cyanine5]GCACAAGATA TGCATCATGG ACGTACACAC TGGAGCC-3′). The following oligonucleotides complementary to human mRNA sequences were used: Fbp1 (5′-[Cyanine5]CTTTAACATG TTCATAACCA GGTCGTTGGA GAGGACGT-3′) and Fbp2 (5′-[Cyanine3]CCTCAGGGAA TTTCTTTTTC TGCACATATC CAGTGGTG-3′). All of the oligonucleotides were synthesized by Sigma–Aldrich. In the controls, the oligonucleotide probes were omitted.

### 2.7. Co-Immunoprecipitation

In the co-immunoprecipitation experiments, the recombinant Fbp2 and Hif1α (Abcam, ab154478) proteins and the Fbp1 isolated from bovine liver were used. The expression and purification of the recombinant human Fbp2 were carried out according to the method described previously [20] with the following modification: in order to obtain a higher yield of the protein, elution was performed twice using a 50 and 100 mM phosphate buffer. The purification of the Fbp1 from a mammalian liver was described previously [21], although here, the incubation with cellulose phosphate (Sigma-Aldrich, c2258) was performed at pH 7.1, and the pH of both the wash and the elution buffer was set to 7.0. The purity of the proteins was assessed via 12% SDS-PAGE and staining the gel with a PageBlue Protein Staining Solution (ThermoFisher, 24620). The absence of proteolysis was confirmed by measuring the ratio of the Fbp enzymatic activity at pH 9.3 and 7.5.

The co-immunoprecipitation reaction was initiated by suspending Hif1α and Fbp1 or Fbp2 proteins (0.25 µM of each protein) in 0.2 mL of buffer containing 0.6 mg/mL BSA and 2 mM MgCl_2_. The mixture was incubated overnight at 4 °C with rotation. Then, the mixture was incubated with the precipitating antibodies (0.5 µM), rabbit anti-Hif1α (Bioss, bs-0737R), or mouse anti-Fbp (produced and tested as described previously [22]), overnight at 4 °C with rotation. After that, 20 µL of the Protein G Agarose (Merck) was centrifuged at 4000× *g* for 2 min, and the precipitates were added to the pelleted Protein G Agarose and incubated overnight at 4 °C with rotation. Then, the precipitates were centrifuged at 4000× *g* for 2 min and washed with PBS. The last step was repeated three times. In the control reactions, the precipitating antibodies were omitted.

The precipitates were then resuspended in the Laemmli’s buffer, resolved by SDS-PAGE (as described below) and a Western Blot analysis was performed with the use of primary antibodies detecting Fbp when the precipitate was obtained with anti-Hif1α antibodies, and detecting Hif1α when the precipitate was obtained using anti-Fbp antibodies.

### 2.8. Western Blot

Cell cultures were homogenized in a buffer containing 100 mM Tris/HCl, 2% SDS, 50 mM DTT, and pH 8.0, and were incubated for 10 min at 99 °C. The homogenates were centrifuged at 20,000× *g* at 4 °C for 20 min, the supernatants were collected, and the total protein concentration was determined using the Bradford’s method. The extracts were resolved using 12% SDS-PAGE and were transferred to the nitrocellulose membrane using a wet system (100 V, 60 min).

After the transfer, the membrane was blocked in 5% BSA in PBS for 1 h and then incubated with primary antibodies diluted in Solution 1 from SignalBoost^TM^ Immunoreaction Enhancer Kit (Millipore, Berlington, MA, USA) overnight at 4 °C. The membrane was washed with 0.1% Triton X-100 in PBS (15 min) and with PBS (15 min) then, it was incubated with secondary antibodies diluted in Solution 2 from the above kit, for 1h at RT. Next, the membrane was washed as described above, and the reaction was visualized using a SuperSignal^TM^ West Pico PLUS Chemiluminescent Substrate (ThermoFisher). The antibodies used in the experiment were as follows: rabbit anti-Hif1α (1:2000, Bioss, bs-0737R), rabbit anti-Fbp2 (1:1000, produced and tested as described previously [19]), mouse anti-Fbp (1:1000, produced and tested as described previously [22]), rabbit anti-β-actin (1:2000, Sigma-Aldrich, a2066), goat anti-rabbit peroxidase-conjugated (1:50,000, Merck, a0545), and goat anti-mouse peroxidase-conjugated (1:50,000, Merck, a9044).

### 2.9. Thermophoresis

The Fbp-Hif1α binding affinity was determined using microscale thermophoresis with the NanoTemper Monolith NT.115 instrument (NanoTemper Technologies GmbH, Munich, Germany). The Hif1α was labeled with a Monolith His-Tag Labeling Kit RED tris-NTA 2^nd^ Generation (Nanotemper Technologies) according to the manufacturer’s instruction. Varying concentrations of Fbp1 and Fbp2 (1.6 µM–0.195 nM and 14 µM–0.427 nM, respectively) were titrated against labeled Hif1α (50 nM) in a PBS buffer supplemented with 0.05% Tween. Samples were loaded into Premium Coated Capillaries (NanoTemper Technologies GmbH), and thermophoresis was measured using 80% LED power and 40% infrared laser power in the ambient temperature of 23 °C. F_0_ and F_1_ times were −1–0 s and 0.5–1.5 s, respectively. Datasets were processed with MO.Affinity Analysis software (NanoTemper Technologies GmbH).

### 2.10. Confocal Microscopy and Fluorescence Analysis

Confocal microscopy analysis was performed as described previously [18]. The images were taken from 10 to 15 randomly selected areas. The quantification of the fluorescence was performed using ImageJ software [23]. Fibroblasts were distinguished from KLN205 and A549 cells by their characteristic morphological features: the longitudinal, spindle-like shape, the regular nuclear contour, the high cytoplasm-nucleus area ratio, and the longitudinal actin filaments parallel to the long axis of the cell [24]. For the analysis of protein and mRNA expression, the edges of the cells were marked using ImageJ software, and the fluorescence of the marked regions was measured.

### 2.11. Statistical Analysis

Data were analyzed using Microsoft Excel 2016. Results are expressed as a mean and standard deviation. In each experiment, at least 30 cells were analyzed. Data were checked for normality using the Shapiro-Wilk test, and for an evaluation of their statistical significance, the Student’s *t*-test was used. A probability of *p* < 0.05 was considered to represent a significant difference.

## 3. Results and Discussion

A recent study has provided a line of evidence for the existence of the reverse Warburg effect in lung cancer. A shift from the oxidative to glycolytic metabolism in normal fibroblasts, and the opposite changes in cancer cells, has been observed when lung fibroblasts were co-cultured with the H1299 lung cancer cells. However, no such changes were observed in the co-culture of fibroblasts and A549 lung cancer cell line [10].

In the present paper, we tested the effect of the co-culturing of lung fibroblasts and lung cancer cells on the expression of Warburg effect-related proteins.

**Figure 1:**
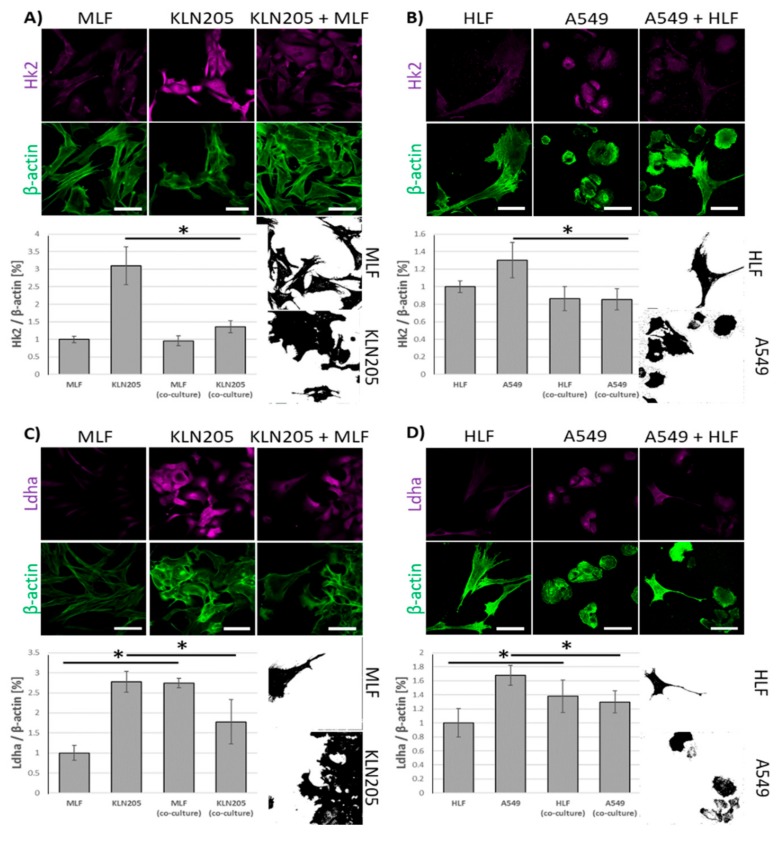
The effect of the co-culturing of cancer cells and fibroblasts on the expression of Hk2 and Ldha. (**A**) and (**B**): the co-culturing of fibroblasts (murine lung fibroblasts (MLF) or human lung fibroblasts (HLF)) with respective cancer cells (KLN205 or A549) decreased the level of Hk2 in cancer cells, having only a minor effect on the enzyme in fibroblasts. (**C**) and (**D**): in the co-cultures, the expression of Ldha was reduced in cancer cells, but was elevated in fibroblasts. Bar = 50 µm. The black and white images depict differences in the shape and size of fibroblasts and cancer cells in the co-cultures. The ratio of the fluorescence of Hk2 and Ldha to β-actin in the MLF and HLF monocultures was assumed to be 1. Asterisks indicate a statistically significant difference (*p* < 0.05). All the experiments were performed in triplicate and obtained similar results, and representative data from one experiment are shown in the figure.

Using fluorescence-based techniques, we found that the co-culturing of murine lung cancer cells (KLN205) with MLF significantly reduced the level of Hk2, a rate limiting enzyme in glycolysis, in the cancer cells, while having only a minor effect on the enzyme in fibroblasts (Figure 1A).

Notably, the co-culturing also resulted in a strong decrease of the fluorescence related to Ldha, the enzyme responsible for lactate synthesis, in the KLN205 cells, elevating its expression in the MLF (Figure 1C). The same pattern of changes in Hk2- and Ldha-related fluorescence was observed when human lung cancer cells (A549) and HLF were co-cultured (Figure 1B,D). Together, the above reorganization of metabolic enzyme expression suggest that, in the co-culture, the cancer cells’ capacity for glucose uptake was reduced, while the fibroblasts’ ability to release lactate was increased.

**Figure 2:**
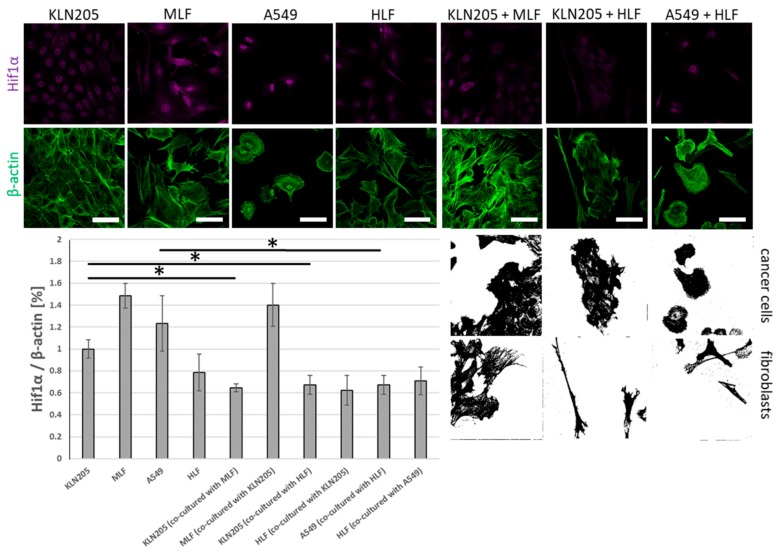
The effect of the co-culturing of cancer cells and fibroblasts on the expression of Hif1α. The co-culturing of murine and human fibroblasts with cancer cells reduced the level of Hif1α in cancer cells, independent of the species’ and fibroblasts’ origins. The effect of cancer cells on the level of Hif1α in fibroblasts was minor. Bar = 50 µm. The black and white images depict differences in the shape and size of the fibroblasts and cancer cells in the co-cultures. The ratio of the fluorescence of Hif1α to β-actin in the KLN205 monoculture was assumed to be 1. Asterisks indicate a statistically significant difference (*p* < 0.05). All the experiments were performed in triplicate with similar results, and representative data from one experiment are shown in the figure.

The observed alterations were associated with the co-culturing-induced reduction in the level of fluorescence related to Hif1α, the main transcriptional regulator of glycolysis, in the KLN205 as well as the A549 cells, after their co-culturing with the respective normal lung fibroblasts (Figure 2). On the other hand, the co-culturing had only a moderate effect on the level of Hif1α in both types of fibroblasts (Figure 2).

Importantly, our data show that the decrease in Hif1α was independent of a source of lung fibroblasts; both the mouse- and the human-derived lung fibroblasts evoked the same changes in the murine cancer cell line (Figure 2).

Together, the alteration in the cellular levels of the three proteins strongly support the hypothesis that the fibroblasts growing in the presence of cancer cells, and presumably also some other cell types (e.g., cardiomyocytes [25]), start to produce lactate while the capacity of cancer cells (and other cells which accompany fibroblasts) to produce lactate is significantly reduced.

Moreover, this also suggests that the metabolic cross-talk between cancer cells and fibroblasts is a common, primeval mechanism in cancer physiology, since it can occur between cells derived from various species.

**Figure 3:**
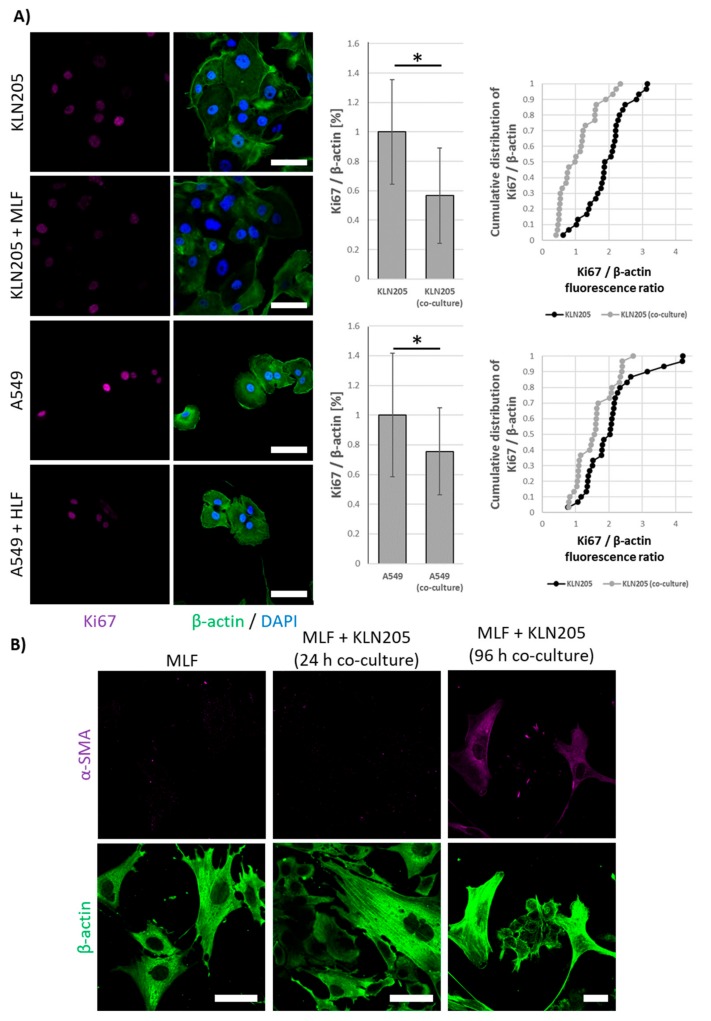
The effect of co-culturing on the proliferative potential of cancer cells and the transformation of fibroblasts into cancer-associated fibroblasts (CAFs). (**A**) 24 h of the co-culturing of fibroblasts and cancer cells lowered the level of Ki67 protein in cancer cells, while the expression of α-SMA in the fibroblasts increased only after 72 h (**B**). Bar = 50 µm. The ratio of the fluorescence of Ki67 to β-actin in the cancer cells monoculture was assumed to be 1. Asterisks indicate a statistically significant difference (*p* < 0.05). All the experiments were performed in triplicate with similar results, and representative data from one experiment are shown in the figure.

Our further studies revealed that the co-culturing of fibroblasts and cancer cells lowered the proliferative potential of both types of cells, as measured by the expression of Ki67 protein (Figure 3A), a cell proliferation marker. This observation corroborates the hypothesis that although fibroblasts are essential for cancer survival, they can inhibit the growth of cancer cells via the “neighbor suppression” ([26,27]; for review see: [13]).

Interestingly, all of the observed changes in the expression of metabolic proteins and Ki67 were very fast and were observed after 24 h of the co-culturing, while an earlier study of Cruz-Bermúdez et al. has shown metabolic modifications in fibroblast and cancer cells only after 4 days of co-culturing [10]. Nonetheless, similar to Cruz-Bermúdez et al., we also observed that the metabolic rearrangement preceded the expression of α-SMA, a protein considered a marker of CAF formation, in fibroblasts (Figure 3B).

The level of Hif1α in cancer cells, has been shown to be inversely proportional to Fbp expression [4], and it has been demonstrated that liver isozyme of Fbp (Fbp1) may interact with Hif1α and direct its degradation [4]. Based on the data, it has been hypothesized that the lowered expression of Fbp1, observed in several cancers (e.g., in clear cell renal cell carcinoma), was responsible for the increased expression of glycolytic enzymes via the stabilization of Hif1α [4].

However, Fbp1 is the main form of Fbp only in gluconeogenic organs, such as the liver and kidney, and, presumably, in cancers derived from these cells, but in other tissues, the muscle Fbp (Fbp2) is predominantly expressed (for review see: [14]).

**Figure 4:**
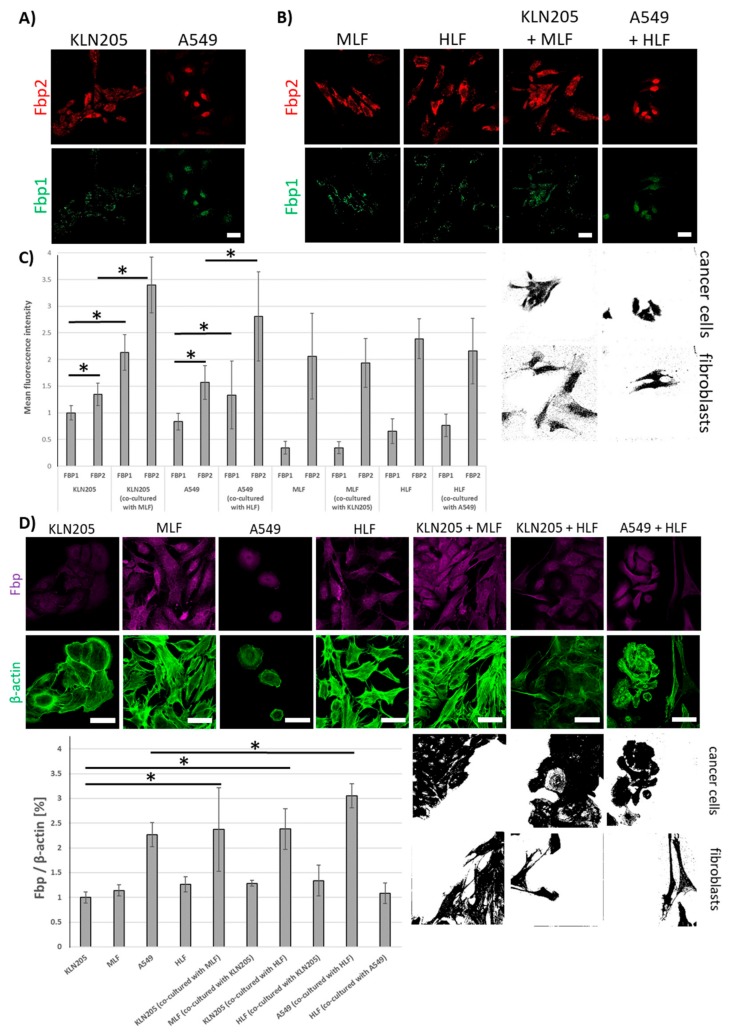
The expression of Fbp in the KLN205 and A549 cells. (**A**) Fbp2 is the main fructose bisphosphatase isoform in murine and human cancer cells. (**B**) the co-culturing of cancer cells with fibroblasts increased the level of mRNA for both Fbp isoforms in cancer cells, having practically no effect on the mRNA levels in fibroblasts. (**C**) the quantification of mRNA expression for Fbp1 and Fbp2 in cancer cells and fibroblasts. (**D**) fibroblast-stimulated changes in the expression of mRNA for Fbp were reflected by an increase in Fbp protein level in cancer cells. Bar = 50 µm. The ratio of the fluorescence of Fbp to β-actin in the KLN205 monoculture was assumed to be 1. Asterisks indicate a statistically significant difference (*p* < 0.05). The black and white images depict differences in the shape and size of the fibroblasts and cancer cells in the co-cultures. All the experiments were performed in triplicate with similar results, and representative data from one experiment are shown in the figure.

The results presented here demonstrate that Fbp2 is also the main form of the enzyme in murine and human cancer cells from the KLN205 and A549 line (Figure 4A,C).

The co-culturing of these cell lines with normal fibroblasts significantly increased levels of mRNAs for both Fbp isozymes (Figure 4B,C) and of Fbp protein-associated fluorescence (Figure 4D) in cancer cells, having practically no effect on Fbp mRNA levels in the fibroblasts (Figure 4C).

To get some insight into the mechanisms of the fibroblasts-dependent induction of Fbp2 expression in cancer cells, we tested the effect of the fibroblast “conditioned medium” on the enzyme expression. Because the action of fibroblasts may be mediated either by the soluble factors of the culture medium or by extracellular vesicles released by fibroblasts, we separately checked the effects of both components on Fbp2 protein level in the A549 cells. The results of our studies reveal that the cancer cells accumulated fibroblasts-derived extracellular vesicles (Supplemental Figure S1A) and that a 24 h incubation with the fibroblasts-derived vesicles significantly stimulated the expression of Fbp2 in cancer cells (Supplemental Figure S1B,C). In contrast to the vesicles, the soluble fraction of the conditioned medium had no effect on Fbp2 level in the A549 cells (Supplemental Figure S1B,C).

This result was not quite surprising, since it has been shown that the protein and nucleic acid cargo of extracellular vesicles, called microvesicles or exosomes [28], shed from fibroblasts mediates cross-talk between them and other cell types, e.g., cancer cells (for review see: [29]).

The regulation of Fbp2 expression has been poorly understood, yet the ability of extracellular vesicles to induce Fbp2 expression restricts the number of hypothetical protein transcription regulators and microRNAs affecting the enzyme level of those present in the vesicles. Thus, the complete quantitative description of the cargo of fibroblast-derived extracellular vesicles is a prerequisite to recognizing the cellular mechanism(s) governing Fbp2 expression.

The ubiquitous expression of Fbp2 (for review see: [14]) and its up-regulation in cancer cells co-cultured with fibroblasts, prompted us to check if Fbp2 may interact with Hif1α and to test the consequences of such hypothetical interactions.

To this end, we performed the co-immunoprecipitation experiments, incubating purified Hif1α with Fbp1 or Fbp2 and precipitating the putative complexes with anti-Hif1α or anti-Fbp antibodies. The precipitates were then resolved, and their components were detected on a Western Blot using one of the antibodies (for details see: Materials and Methods).

**Figure 5:**
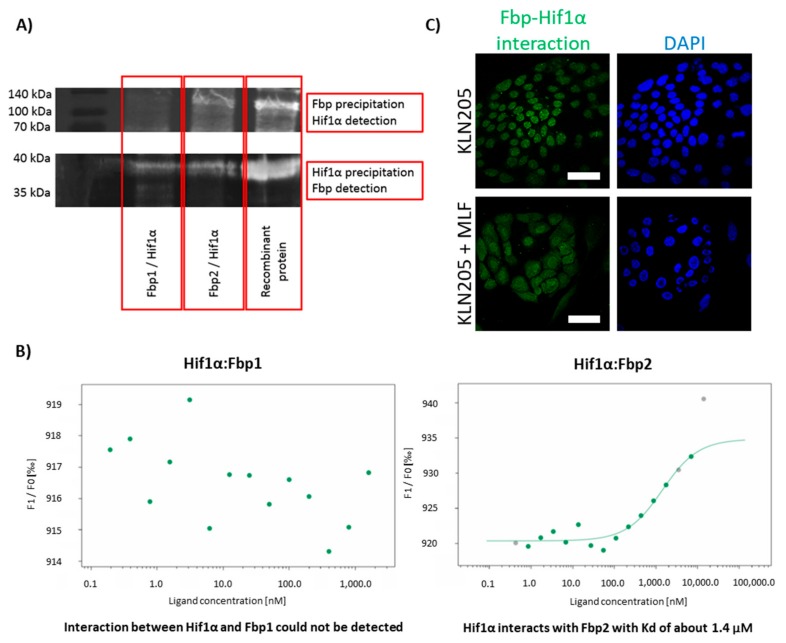
Fbp2 is a binding partner of Hif1α in vitro and in cell cultures. (**A**) co-immunoprecipitation of the Hif1α-Fbp complex; the upper image shows the Western Blot detection of the Hif1α protein after the precipitation of the complex with anti-Fbp antibodies, while the lower shows the immunodetection of Fbp after the precipitation with the use of anti-Hif1α IgG. (**B**) Hif1α binds to Fbp2 (Kd = 1.4 µM) but not to Fbp1, as quantified by the microscale thermophoresis experiments. (**C**) Fbp’s association with Hif1α in the KLN205 cells monoculture, was restricted to the cells’ nuclei (upper images), whereas in the co-culture of the KLN205 and fibroblasts, the Hif1α-Fbp2 complex-associated signal was uniformly dispersed in the nuclei and cytoplasm of the cancer cells (lower images). Bar = 50 µm. All the experiments were performed in triplicate with similar results, and representative data from one experiment are shown in the figure.

Upon probing the blot membrane that was carrying proteins from the precipitates obtained with anti-Hif1α antibodies, we observed the Fbp-related signal regardless of the used Fbp isoform (Figure 5A). However, upon probing the membrane that was carrying proteins from the precipitates obtained with the anti-Fbp antibodies, we could detect a substantial Hif1α-related signal only if Fbp2 was in the initial precipitate. This suggests that the Hif1α-Fbp1 interaction was much weaker than the Hif1α-Fbp2 one.

Therefore, to get some deeper insight into this interaction, we used the thermophoresis technique to measure Hif1α affinity to Fbp isozymes. The results confirm that Fbp2 bound to Hif1α with relatively high affinity (the dissociation constant, Kd = 1.4 µM), while the interaction of Fbp1 with Hif1α was very weak, almost undetectable (Figure 5B). However, such a weak interaction does not rule out the downregulation of Hif1α levels by Fbp1 in gluconeogenic tissues, where the expression of Fbp1 is relatively high [30], and thus, in spite of the very low affinity between Fbp1 and Hif1α, Fbp1 may significantly saturate Hif1α, directing it to degradation.

Our further studies using the DuoLink technique revealed that the association of Fbp with Hif1α in the KLN205 cells monoculture was restricted to the cells’ nuclei (Figure 5C). This was quite unexpected since the exclusively nuclear location of the Fbp-Hif1α complex did not harmonize well with the putative Fbp-directed degradation of Hif1α, which should take place in the cytoplasm.

In fact, this suggested that the formation of the Hif1α-Fbp2 complex in the nuclei of the KLN205 cells, which rely on the glycolytic metabolism, was responsible for the stabilization of the transcriptional activity of Hif1α rather than degradation of the protein.

In contrast, in the co-culture of the KLN205 cells and fibroblasts, while the expression of Hif1α and glycolytic enzymes was decreased in the cancer cells, the Hif1α-Fbp2 complex-associated signal was dispersed uniformly throughout the nuclei and cytoplasm of these cells (Figure 5C). Such a cytoplasmic localization of the complex might point to the previously suggested Fbp-guided proteolysis of Hif1α.

**Figure 6:**
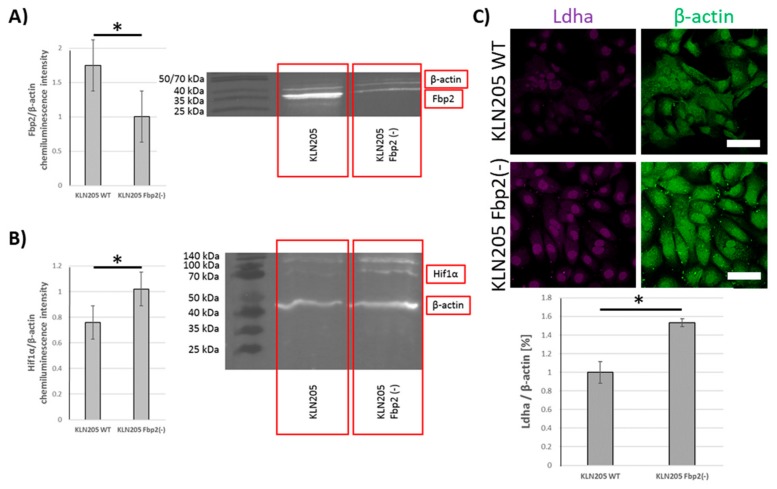
Fbp2 silencing in cancer cells stimulated expression of glycolytic proteins. (**A**) Fbp2 silencing resulted in Hif1α (**B**) and Ldha elevation (**C**) in the KLN205 cells. Bar = 50 µm. Asterisks indicate a statistically significant difference (*p* < 0.05). All the experiments were performed in triplicate with similar results, and representative data from one experiment are shown in the figure.

Since the experiments were performed using antibodies that were unable to distinguish between Fbp isozymes (because of the high similarity of Fbp isoforms it is essentially not possible to obtain antibodies that are able to distinguish between the two physiological, globular forms of Fbp isozymes) we tested whether the levels of Ldha and Hif1α in cancer cells depend on Fbp2 amount or the consequences of Fbp2-Hif1α interactions that are not related to glycolysis. It has been previously shown that, in cancer cells, the overexpression of Fbp1 and Fbp2, respectively, stimulated Hif1α degradation [31], suppressed glucose metabolism, and reduced cell proliferation [32]. Our results reveal that, in the KLN205 cells, the partial (about 50%) silencing (Figure 6A) of Fbp2 expression with shRNA very significantly increased Hif1α and Ldha levels (Figure 6B,C). This is a strong piece of evidence supporting the notion that Fbp2 is a crucial factor regulating the glycolytic capacity of lung cancer cells. Taking into account that Fbp2 is broadly expressed in the whole organism, it may be hypothesized that the isoform may also be the main modulator of Hif1α-dependent transcription in the majority of cells and organs.

Together, the results presented in this paper demonstrate that the co-culturing of lung fibroblasts and lung cancer cells, even if the cells were derived from different species, reduced glycolytic capacity of the cancer cells and stimulated lactate production in the fibroblasts, thus pointing to emergence of the reverse Warburg effect in the co-cultures. We suggest that this results from the Fbp-mediated degradation of Hif1α, the main regulator of glycolytic gene expression. Additionally, we imply that, except from the gluconeogenic organs and cancers derived from them, Fbp2, but not Fbp1, is responsible for the Hif1α degradation. On the other hand, Fbp2 in a cell nucleus may stabilize Hif1α and induce the Hif1α-dependent transcription of metabolic genes. This is in line with previous findings showing the presence of Fbp2 in the nuclei of cells with high glycolytic rates [19].

This dual role of Fbp2— the stabilization of the Hif1α transcriptional activity in the nucleus and the promotion of the transcription factor for the degradation in cytoplasm—may result from unique structural and kinetic properties of the isozyme. Fbp2 is over an order of magnitude that is more sensitive than Fbp1 to allosteric inhibitors, AMP, and NAD^+^ (for review see [14]). In contrast to Fbp1, Fbp2 is also strongly inhibited by calcium ions [33].

Fbp2 exists as a mixture of dimers and tetramers, and in a living cell, the oligomeric state of Fbp2, modulated by its allosteric inhibitors, affects the ability of the enzyme to interact with cellular components [20]. Moreover, in contrast to Fbp1, which is a planar tetramer, Fbp2, in its active tetrameric conformation, adopts a unique cruciform-like quaternary structure, in which completely new protein surfaces are exposed and become targets for interactions with various binding partners [20].

All of the above-mentioned unique features of Fbp2 make the isoform an ideal physiological-conditions-controlled “switch” for cellular metabolism.

## 4. Conclusions

The results of our studies, with the use of lung fibroblasts and lung cancer cells, concur with the hypothesis that the reciprocal interactions of these cells induce the so-called reverse Warburg effect. Furthermore, they suggest that the hypoxia/Hif1α-dependent regulation of the metabolism in cancer is modulated through a Fbp2-dependent sensing of the energy and redox state of a cell. Finally, they also show that the metabolic cross-talk between cancer cells and fibroblasts is a common, primeval mechanism in cancer biology since it can occur even if the two cell types originate from different species.

## Supplementary Materials

The following are available online at https://www.mdpi.com/2073-4409/9/1/205/s1, Figure S1: The effect of fibroblast-conditioned medium on Fbp expression in the A549 cancer cells.
