# Supplementary material for: The Reverse Warburg Effect Is Associated with Fbp2-Dependent Hif1α Regulation in Cancer Cells Stimulated by Fibroblasts
Source: Cells. 2020 Jan 14;9(1):205. doi: 10.3390/cells9010205 (PMC7016812; doi:10.3390/cells9010205)
Supplement: Supplementary file 1 [file cells-09-00205-s001.pdf]

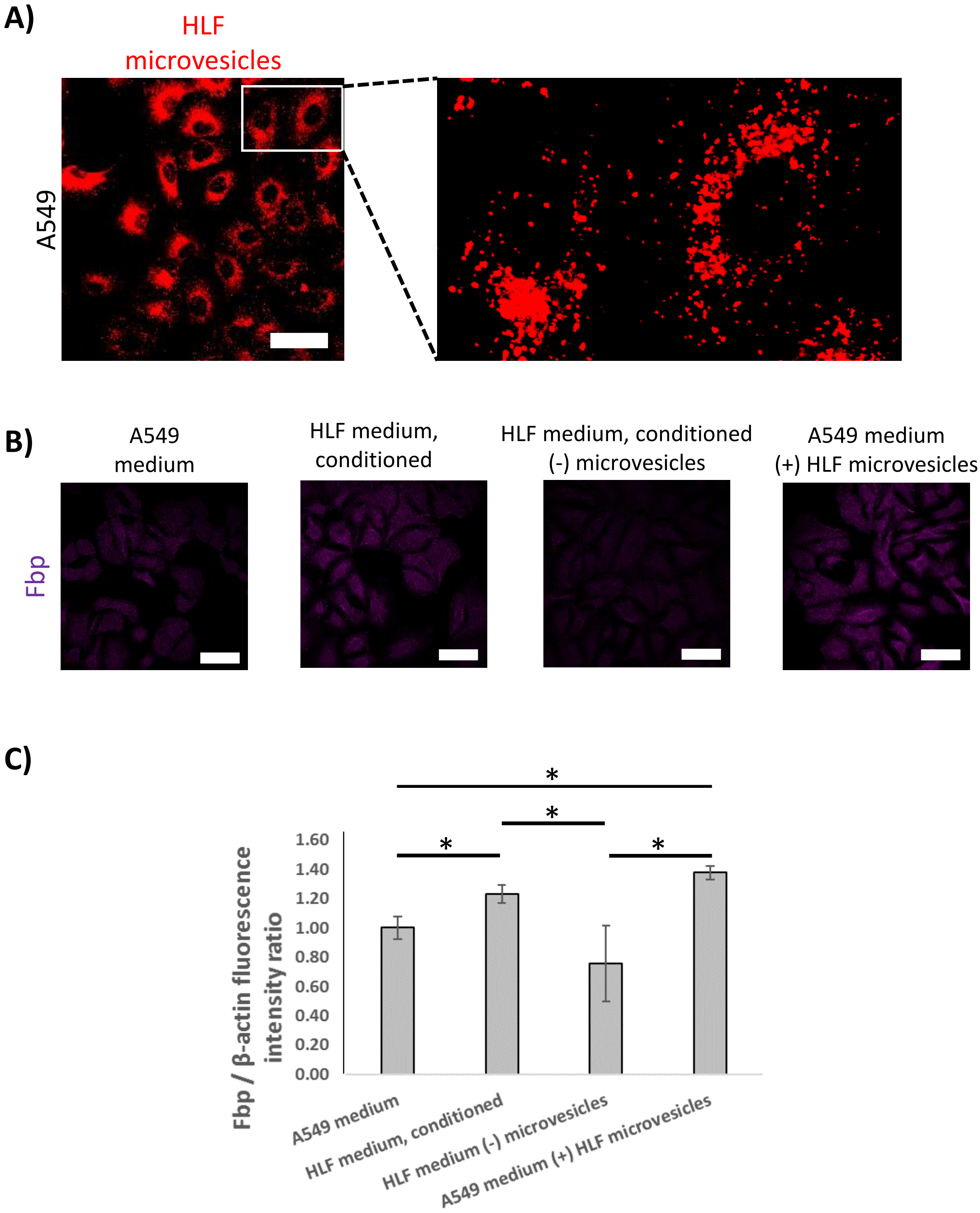


**Supplementary Figure 1**

**The effect of fibroblast-conditioned medium on Fbp expression in the A549 cancer cells**

A) A549 cells absorb the fibroblast-derived microvesicles isolated from the medium obtained from the 48h culture of HLF cells (so-called “conditioned medium”).

B) The microvesicle cargo increases the amount of Fbp in the cancer cells, in contrast to the components of the microvesicle-deprived conditioned medium (C).

Bar=50 µm. The results are expressed as mean and standard deviation. Asterisks indicate a statistically significant difference ($p < 0.05$).

All the experiments were performed in triplicate with similar results and representative data from one experiment is shown in the figure.
